# Mobile phone bacterial contamination among students and staff at Haramaya University, Eastern Ethiopia: A cross-sectional study

**DOI:** 10.1097/MD.0000000000050780

**Published:** 2026-09-25

**Authors:** Firayad Ayele, Kedir Urgesa, Shambel Mekonnen, Kasahun Bogale, Mohammed Ahmed, Sileshi Debebe, Fikru Tebeje, Haftu Asmerom, Mesay Arkew, Tadesse Shume, Tewodros Tesfa, Fitsum Weldegebreal

**Affiliations:** a School of Medical Laboratory Sciences, College of Health and Medical Sciences, Haramaya University, Harar, Ethiopia.

**Keywords:** antimicrobial susceptibility pattern, bacterial contamination, bacterial isolates, Ethiopia, Harar, medical students, mobile phone

## Abstract

Mobile phones are used by most of the global population, particularly by college and university students and healthcare professionals. Despite their benefits, potential health risks associated with mobile-phone contamination may be overlooked. Therefore, this study aimed to determine bacterial contamination, associated factors, and antimicrobial susceptibility patterns of bacterial isolates from mobile phones among students and employees of Haramaya University College of Health and Medical Sciences, Harar, Eastern Ethiopia. An institution-based cross-sectional study was conducted among 238 students and employees from July 1 to August 30, 2022. Participants were selected using stratified sampling techniques. A structured questionnaire was used to collect the data. A swab sample was collected from each participant’s mobile phone using a sterilized cotton swab and transported to the laboratory within 2 hours for microbiological investigation. Bacterial isolates were identified using colony morphology, gram staining, and biochemical tests. Antimicrobial susceptibility testing was performed using the Kirby–Bauer disk diffusion method. Bivariable and multivariable logistic regression analyses were used to identify factors associated with bacterial contamination. Bacterial contamination was detected on 89.5% (95% confidence interval [CI]: 87–92) of sampled mobile phones. A total of 235 bacterial isolates were recovered, of which *Staphylococcus epidermidis* was the predominant isolate (70.6%), followed by *Staphylococcus saprophyticus* (14.9%) and *Staphylococcus aureus* (6.4%). Among the bacterial isolates tested for antimicrobial susceptibility, susceptibility was highest to gentamicin and chloramphenicol (94.0% each), followed by clindamycin (83.4%). Resistance was highest to ampicillin (98.7%), kanamycin (88.2%), cefotaxime (71.5%), cefoxitin (54.9%), and ciprofloxacin (52.8%). Overall, 181 (77.02%) isolates were multidrug-resistant, and 25 (10.64%) were extensively drug-resistant. Male sex (adjusted odds ratio [AOR]: 3.92, 95% CI: 1.364–11.282), lack of regular mobile-phone cleaning (AOR: 7.25, 95% CI: 2.161–24.336), and lack of awareness about mobile-phone bacterial contamination (AOR: 4.25, 95% CI: 1.293–13.970) were independently associated with bacterial contamination. This study demonstrated a high level of bacterial contamination of mobile phones among students and employees. *S epidermidis* was the predominant bacterial isolate. High levels of antimicrobial resistance were observed, particularly to ampicillin, kanamycin, and cefotaxime. The findings support the need to promote regular mobile-phone cleaning and improve awareness of mobile-phone hygiene.

## 1. Introduction

Mobile phones and computers are an indispensable part of people’s daily lives worldwide.^[1]^ Smart technologies are widely used by the majority of the world’s population due to their wide range of uses and benefits, particularly by college and university students and healthcare professionals for communication and access to information.^[2,3]^ However, the frequent handling and close personal contact associated with mobile phones have raised concerns regarding their potential to harbor microorganisms and serve as fomites for microbial transfer.

Recent studies have demonstrated that mobile phones can harbor a wide range of infectious microorganisms, including community-acquired bacterial pathogens, due to repeated contact with contaminated hands, environmental surfaces, and human secretions.^[4–8]^ However, due to their various benefits, health-related concerns associated with contamination are often overlooked.^[9]^ Many individuals perceive microorganisms as being restricted to laboratories, hospitals, or clinical environments, resulting in a false sense of safety in daily settings.^[9]^ However, bacteria are ubiquitous and can survive on inanimate surfaces, which may serve as reservoirs for the persistence and transfer of microorganisms. The repeated use of mobile phones without adequate cleaning or disinfection may therefore provide opportunities for bacterial contamination and potential transfer between individuals and environmental surfaces in both community and institutional settings.^[10]^

Healthcare-associated infections remain a major global public health concern. It is estimated that approximately 1.7 million healthcare-associated infections caused by different microorganisms occur annually in the United States, contributing to nearly 99,000 deaths.^[11]^ Previous studies have reported considerable bacterial contamination of mobile phones among different population groups, with some studies indicating higher contamination rates among students compared with healthcare professionals.^[12]^ A study conducted among students at the University of Cape Coast, Ghana, found that all sampled mobile phones were contaminated with various bacterial species, many of which demonstrated resistance to commonly used antibiotics.^[3]^ Similarly, several studies have reported that mobile phones may harbor bacteria with clinically important antimicrobial resistance profiles.^[3,13]^

Smartphones are controlled by glass touchscreens that come into direct contact with the fingertips, making them potential carriers of microorganisms present on the hands.^[14]^ The frequent handling of mobile phones and their repeated contact with hands and environmental surfaces may facilitate the accumulation and persistence of microorganisms on their surfaces.^[15]^ However, the detection of bacteria on mobile phones does not, by itself, establish the source or timing of contamination and cannot demonstrate healthcare-associated or nosocomial acquisition. Contamination may originate from healthcare-associated environments, community environments, personal hygiene practices, or other sources.

Antimicrobial agents are used to prevent infection by pathogens that are susceptible to them.^[16]^ Antibiotic drug resistance has now become a worldwide problem, resulting in increased morbidity and mortality, high treatment failure rates, and higher healthcare expenditures.^[1]^ Some pathogens have developed resistance to numerous medications, and resistant bacterial infections have become all too common.^[16]^ Furthermore, bacteria isolated from mobile phones have been found to be multidrug-resistant (MDR), according to investigations.^[17]^ A study that looked at the frequency and antimicrobial susceptibility pattern of bacteria isolated from healthcare professionals’ mobile phones found that 17% of the isolates were resistant to routinely used antibiotics.^[3]^

Although previous studies have investigated bacterial contamination of mobile phones, most have focused on healthcare workers, with limited attention given to health science students and institutional employees who frequently interact with healthcare environments. These groups represent a unique population because they combine characteristics of both community members and future or current healthcare personnel, potentially increasing opportunities for exposure to and transfer of microorganisms from different sources. However, interaction with healthcare environments does not necessarily indicate healthcare-associated acquisition or nosocomial transmission. Furthermore, evidence regarding bacterial contamination, antimicrobial susceptibility patterns, and associated factors among mobile phone users in Ethiopian health science institutions remains limited.

Therefore, there is a significant knowledge gap regarding the extent of bacterial contamination and antimicrobial resistance among bacteria isolated from mobile phones of students and employees in health science institutions. Understanding the extent of contamination, associated factors, and resistance patterns is important for developing appropriate mobile phone hygiene and infection prevention practices. Therefore, this study aimed to determine bacterial contamination, associated factors, and antimicrobial susceptibility patterns of bacterial isolates from mobile phones among students and employees of Haramaya University College of Health and Medical Sciences (HU-CHMS), Harar, Eastern Ethiopia.

## 2. Methods and materials

### 2.1. Study design, period, and Area

A facility-based cross-sectional study was conducted from July 1 to August 30, 2022, at the HU-CHMS, Harar, Eastern Ethiopia. HU-CHMS includes a teaching hospital that provides healthcare services and serves as a training site for undergraduate and postgraduate students. At the time of the study, the total source population was 3115, comprising 1615 employees and 1500 students (https://www.haramaya.edu.et).

### 2.2. Study participants and selection criteria

The source population of this study consisted of all students and employees of HU-CHMS, Harar, Eastern Ethiopia, who owned and used a mobile phone during the study period. The study population included all eligible individuals from the HU-CHMS source population. Participants were eligible for inclusion if they were students or employees of HU-CHMS, owned a mobile phone, were available during the study period, volunteered to participate, and provided written informed consent. Individuals who were unwilling to participate or unable to provide access to their mobile phones for sample collection were excluded from the study.

### 2.3. Sample size determination and sampling technique

The sample size was calculated using the single population proportion formula. The calculation was based on a previously reported prevalence of bacterial contamination of mobile phones (94.2%),^[16]^ a 95% confidence level, a 3% margin of error, and a 10% nonresponse rate. Since the total population size of HU-CHMS was <10,000, a finite population correction formula was applied. Accordingly, the final sample size was determined to be 238 participants.

A stratified sampling technique was employed to ensure proportional representation of employees and students. The total source population (n = 3115) was stratified into employees (n = 1615) and students (n = 1500). Based on proportional allocation, 123 participants were selected from employees and 115 from students, giving a total of 238 participants. Participants within each stratum were selected using a simple random sampling technique. Mobile phone samples were collected from selected participants who provided written informed consent.

### 2.4. Data collection and sample collection procedures

A brief, self-administered, pretested questionnaire adapted from previously published literature^[16–18]^ was used to collect sociodemographic characteristics and information on potential risk factors associated with bacterial contamination of mobile phones. Written informed consent was obtained from each participant before questionnaire administration and sample collection.

After the completion of self-administered questionnaires, a swab sample was collected from the participant’s mobile phone. Before taking a swab, both hands of the data collectors were cleaned using an alcohol-based instant hand sanitizer, and powder-free disposable gloves were worn per sample throughout the work to prevent cross-contamination. A sterilized cotton swab moistened with sterile normal saline was rotated to swipe the overall area of the mobile phone. In the case of mobile phones with covers, the swab was taken from the outer surfaces of the cover, besides the screen. Then, the swab was placed immediately into sterile normal saline in a sterile container and transported to the Haramaya University Microbiology Laboratory with correct and complete labeling within 30 minutes for microbiological analysis.

### 2.5. Sample processing and antimicrobial susceptibility testing

The collected samples were inoculated onto Blood Agar and MacConkey Agar using the standard streak-plate technique. The inoculated plates were incubated aerobically at 37°C for 24 to 48 hours. Primary bacterial isolation was performed based on colony morphology and Gram staining.^[19]^ Further bacterial identification was performed using appropriate biochemical tests, including triple sugar iron agar, indole, citrate, oxidase, urease, motility, Voges–Proskauer, methyl red, mannitol, catalase, and coagulase tests, as appropriate for the suspected bacterial isolates.^[19]^

### 2.6. Antimicrobial susceptibility testing

Antimicrobial susceptibility testing was conducted using the Kirby Bauer disc diffusion method according to Clinical and Laboratory Standard Institute guidelines.^[20,21]^ A bacterial suspension equivalent to a 0.5 McFarland turbidity standard was prepared and uniformly inoculated onto Mueller–Hinton agar plates using a sterile cotton swab. The antimicrobial disks used for susceptibility testing included gentamicin (10 µg), ampicillin (10 µg), chloramphenicol (30 µg), trimethoprim–sulfamethoxazole (1.25/23.75 µg), cefoxitin (30 µg), doxycycline (30 µg), erythromycin (15 µg), clindamycin (2 µg), kanamycin (30 µg), ciprofloxacin (5 µg), and cefotaxime (30 µg). These antimicrobial disks were selected according to the bacterial species isolated and the recommendations of the 2020 Clinical and Laboratory Standard Institute (CLSI) guidelines. Following incubation, the inhibition zones were measured and interpreted according to the CLSI interpretive standards.^[20]^ Bacterial isolates were categorized as susceptible, intermediate, or resistant according to the applicable CLSI breakpoints.^[20]^

### 2.7. Data quality assurance

The questionnaire was 1st translated from English into the local languages (Amharic and Afan Oromo) by linguists, and then it was translated back into English by additional linguists. To ensure its consistency and clarity prior to the actual data collection, 5% of the questionnaire underwent pretesting at the Haramaya University main campus. The lead investigator provided training for data collectors and laboratory personnel prior to the start of the actual data collection. The sterility of the culture medium was checked by incubating it at 35°C–37°C for an overnight period without specimen inoculation. Additionally, the performance of the culture medium and antibiotic disks was evaluated using reference strains of *Escherichia coli* (American Type Culture Collection [ATCC] 25922), *Pseudomonas aeruginosa* (ATCC 27853), and *Staphylococcus aureus* (ATCC 25923).^[20]^ The turbidity of the bacterial suspension’s turbidity was adjusted to achieve the 0.5 McFarland level to be able for performing a drug susceptibility test.

### 2.8. Definition of bacterial contamination

A mobile phone was considered bacterially contaminated when 1 or more bacterial species were isolated from the sampled mobile-phone surface. Verify that this was the operational definition used in your original study.

### 2.9. Definition of multidrug resistance

An isolate was classified as MDR when it demonstrated acquired non-susceptibility to at least 1 antimicrobial agent in 3 or more antimicrobial categories. An isolate was classified as extensively drug-resistant (XDR) when it remained susceptible to only 1 or 2 antimicrobial categories, and pandrug-resistant when it was non-susceptible to all antimicrobial agents in all antimicrobial categories tested. These definitions should be retained only if they match the definitions actually used in your original study.

### 2.10. Statistical analysis

Data were entered into EpiData version 4.6 (Odense, Denmark) and exported to SPSS version 24 (IBM Corp.) for statistical analysis. Descriptive statistics, including frequencies, proportions, and appropriate measures of central tendency, were used to summarize the study variables.

Bivariable logistic regression analysis was performed to identify candidate variables associated with bacterial contamination of mobile phones. Variables with a *P* value <.25 in the bivariable analysis were considered for inclusion in the multivariable logistic regression model. Adjusted odds ratios (AORs) with corresponding 95% confidence intervals (CIs) were calculated to assess the strength and precision of associations. Statistical significance in the final multivariable logistic regression model was declared at a *P* value <.05.

### 2.11. Ethical consideration

Haramaya University’s College of Health and Medical Science’s Institutional Health Research Ethics Review Committee provided ethical approval. An official letter of support was sent to all of the college’s schools, departments, and administration offices. The informed, voluntary, written, and signed consent was signed by the college’s executive director. Participants provided informed, voluntary, written, and signed consent. The methodology and relevance of the study were explained to the study participants. Any information regarding the data was kept private, and the outcome was only communicated to an authorized organization. Every study participant had the right to refuse to participate in the study, and those who did not wish to participate were not forced to do so.

## 3. Results

### 3.1. Characteristics of participants

A total of 238 participants were included in the study. More than half of the participants were female (54.2%). The majority were aged 20 to 29 years (69.3%), with a mean age of 28.9 years. Most participants were single (161, 67.6%), and 182 (76.5%) had a Bachelor of Science degree or equivalent 1st degree. By occupational category, 116 (48.7%) participants were students, 70 (29.4%) were administrative employees, 27 (11.3%) were lecturers, 16 (6.7%) were food handlers, and 9 (3.8%) were cleaners (Table 1).

**Table 1:** Sociodemographic characteristics of HU-CHMS students and employees, Harar, Eastern Ethiopia, 2022 (n = 238).

Variable	Categories	Frequency (N)	Percent (N %)
Sex	Male	115	48.3
	Female	123	51.7
Age	20–29	165	69.3
	30–39	52	21.8
	40–49	12	5
	50–59	8	3.4
	60–69	1	0.4
Marital status	Single	161	67.6
	Married	75	31.5
	Divorced	2	0.8
Educational status	Primary	24	10.1
	Secondary	26	10.9
	Preparatory	6	2.5
	Higher education	182	76.5
Occupational status	Employee/employee	122	51.3
	Student with clinical attachment	74	31.1
	Student without clinical attachment	42	17.6
Working area	Administrative employee	70	29.4
	Cleaner	9	3.8
	Food handler	16	6.7
	Lecturer	27	11.3

HU-CHMS = Haramaya University College of Health and Medical Sciences.

### 3.2. Mobile phones and infection prevention

Most participants (82.8%) used touchscreen mobile phones, while 31.3% did not have protective phone covers. Approximately 13.0% of participants had owned their mobile phones for <6 months. More than half of the participants reported sharing their mobile phones with other individuals, and 46.2% reported using their mobile phones in the toilet. Regarding phone-cleaning practices, 15.5% of participants reported regularly cleaning their mobile phones, while 22.8% used alcohol for disinfection. Although 54.6% of participants believed that mobile phones could carry bacteria, the majority reported that they did not wash their hands after using their mobile phones (Table 2).

**Table 2:** Characteristics on the use of mobile phones and infection prevention of HU-CHMS students and employees, Harar, Eastern Ethiopia, 2022 (n = 238).

Variables	Categories	Frequency (N)	Percent (N %)
Types of mobile phone	Touch screen	197	82.8
	Keypad	41	17.2
Age of mobile phone	<6 mo	31	13
	>6 mo	34	14.3
	>12 mo	173	72.7
Presence of mobile phone cover	Yes	152	63.9
	No	86	36.1
Mobile phone sharing	Yes	134	56.3
	No	104	43.7
Using mobile phone in the toilet	Yes	110	46.2
	No	128	53.8
Frequency of cleaning mobile phone	Daily	37	15.5
	Weekly	64	26.9
	Never	137	57.6
Ways of cleaning mobile phone	Using alcohol	23	22.8
	Using other product	78	77.2
Awareness on contamination of mobile phone by pathogens	Yes	130	54.6
	No	108	45.4
Hand washing habit after using mobile phone	Yes	42	17.6
	No	196	82.4

Using other product (soap, wipe, cloth, or paper).

HU-CHMS = Haramaya University College of Health and Medical Sciences.

### 3.3. Prevalence and type of bacterial isolates

The overall prevalence of bacterial contamination among the swabbed mobile phones was 89.5% (95% CI: 87%–92%). Approximately 11.3% of the contaminated phones showed contamination with multiple bacterial species, and bacterial isolates were identified based on phenotypic characteristics. Gram-positive bacteria were the predominant isolates, with *Staphylococcus epidermidis* (70.6%) being the most frequently isolated bacterium, followed by *Staphylococcus saprophyticus* (14.9%), *S aureus* (6.4%), and *Micrococcus* spp. (2.6%). Other bacterial isolates included *Klebsiella aerogenes* (1.3%), Gram-positive rods (0.9%), Gram-negative cocci (0.8%), *Klebsiella pneumoniae* (0.7%), and *Enterobacter aerogenes* (0.9%) (Fig. 1).

**Figure 1.**
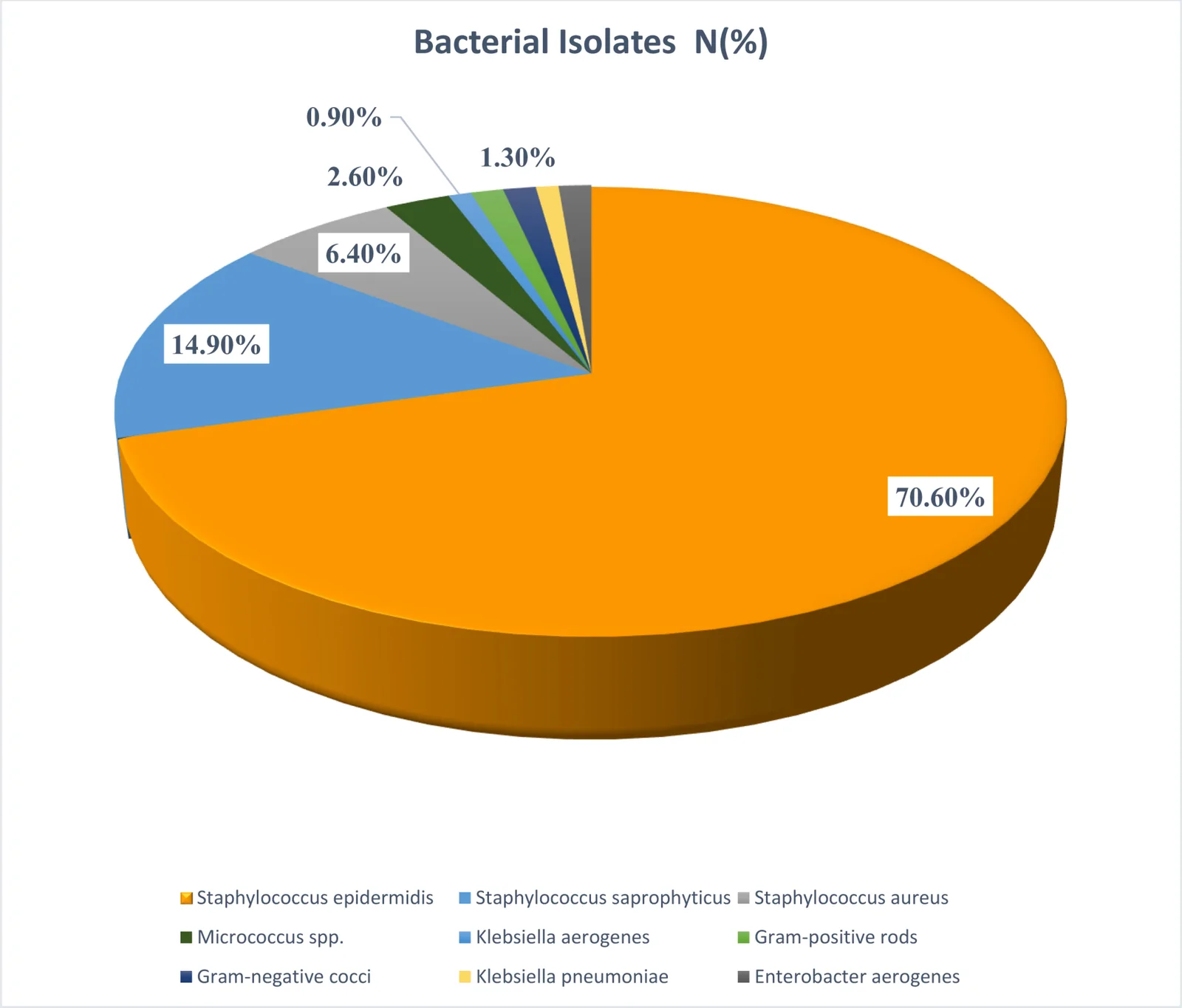
Distribution of bacterial isolates recovered from mobile phones of students and employees at Haramaya University College of Health and Medical Sciences, Harar, Eastern Ethiopia, 2022.

The prevalence of mobile phone contamination was higher in the age group of 20 to 29 years (67.6%). All of the mobile phones carried by food handlers and cleaners were contaminated with bacterial isolates. The mobile phones owned by 97.5% of the study participants who had not cleaned their mobile phones at all had bacterial contamination, whereas 90.3% of study participants with no habit of cleaning their hands after using mobile phones had bacterial isolates from the phone. The prevalence of bacterial contamination of phones was about 96.3%, 92.7%, 92.3%, and 90% amongst study participants who were not aware of contamination of mobile phones by pathogens, had keypad phones, cleaned their mobile phones using other products (soap, wipes, cloth, or paper) rather than alcohol, and had a habit of using their mobile phones while confined in the toilet, respectively.

### 3.4. Factors associated with mobile phone contamination

In the bivariate analysis, sex, occupation, frequency of mobile phone cleaning, and awareness of mobile phone contamination were considered candidate variables for the multivariable logistic regression model based on a screening criterion of *P* < .25. Male sex, weekly or no mobile phone cleaning, and lack of awareness of mobile phone contamination were significantly associated with bacterial contamination in the bivariate analysis.

In the multivariable analysis, male participants had significantly higher odds of bacterial contamination of their mobile phones than female participants (AOR: 3.92, 95% CI: 1.36–11.28, *P* = .011). Participants who never cleaned their mobile phones had >7 times higher odds of bacterial contamination than those who cleaned their phones daily (AOR: 7.25, 95% CI: 2.16–24.34, *P* = .001). Similarly, participants who were unaware that mobile phones could be contaminated with bacteria had significantly higher odds of bacterial contamination than those who were aware (AOR: 4.25, 95% CI: 1.29–13.97, *P* = .017). After adjustment for the other variables in the model, occupation and weekly mobile phone cleaning were not significantly associated with bacterial contamination (Table 3).

**Table 3:** Factors associated with bacterial contamination of mobile phones among students and employees of HU-CHMS, Harar, Eastern Ethiopia, 2022.

Variable	Characteristics	Mobile phone bacterial contamination, n (%)	COR (95% CI)	*P* value	AOR (95% CI)	*P* value
Sex	Female	117 (95.1)	1	–	1	–
	Male	96 (83.5)	0.259 (0.119–0.564)	.001	3.923 (1.364–11.282)	.011
Occupation	Employee	113 (92.6)	2.093 (0.675–6.495)	.201	0.418 (0.102–1.715)	.226
	Student with clinical attachment	64 (86.5)	1.067 (0.328–3.468)	.915	0.722 (0.184–2.832)	.640
	Student without clinical attachment	36 (85.7)	1	–	1	–
Frequency of phone cleaning	Daily	27 (73.0)	1	–	1	–
	Weekly	58 (90.6)	3.580 (1.211–10.589)	.021	1.486 (0.455–4.855)	.512
	Never	128 (93.4)	5.267 (1.954–14.199)	.001	7.252 (2.161–24.336)	.001
Awareness of phone contamination	Yes	109 (83.8)	Ref.	1	1	1
	No	104 (96.3)	5.009 (1.663–15.086)	.004	4.249 (1.293–13.970)	.017

The crude ORs and 95% CIs were recalculated from the frequencies presented in the table. AORs were retained from the original analysis and should be verified against the original multivariable logistic regression output before submission.

AOR = adjusted odds ratio, CI = confidence interval, COR = crude odds ratio, HU-CHMS = Haramaya University College of Health and Medical Sciences, Reference category = 1.

### 3.5. Antimicrobial susceptibility patterns of bacterial isolates

Antimicrobial susceptibility testing was performed on 235 bacterial isolates. Gentamicin and chloramphenicol showed the highest proportions of susceptible isolates, with 221 (94.0%) isolates susceptible to each antimicrobial, followed by clindamycin (196, 83.4%), trimethoprim–sulfamethoxazole (173, 73.6%), and erythromycin (163, 69.4%). In contrast, the highest proportions of resistance were observed for ampicillin (232, 98.7%), kanamycin (205, 88.2%), cefotaxime (168, 71.5%), cefoxitin (129, 54.9%), and ciprofloxacin (124, 52.8%).

Among the predominant isolates, *S epidermidis* and *S aureus* showed high susceptibility to gentamicin (95.8% and 93.3%, respectively) and chloramphenicol (97.6% and 100%, respectively). *S saprophyticus* showed high susceptibility to gentamicin (97.1%) but lower susceptibility to chloramphenicol (82.9%). The 3 predominant *Staphylococcus* species showed high resistance to ampicillin (98.2%–100%) and kanamycin (85.7%–93.3%). Gram-negative isolates, including *P aeruginosa*, *K pneumoniae*, and *E aerogenes*, showed high resistance to ampicillin and cefotaxime, although susceptibility patterns varied across the other tested antimicrobials (Table 4).

**Table 4:** Antimicrobial susceptibility patterns of bacterial isolates recovered from the mobile phones of students and employees at Haramaya University College of Health and Medical Sciences (HU-CHMS), Harar, Eastern Ethiopia, 2022.

Bacterial isolate	Pattern	GM	AMP	CHL	SXT	CXT	DXT	E	CLN	K	CIP	CXM
*Staphylococcus epidermidis* (n = 166)	S	159 (95.8%)	3 (1.8%)	162 (97.6%)	118 (71.1%)	82 (49.4%)	106 (63.9%)	123 (74.1%)	144 (86.7%)	19 (11.5%)	83 (50.0%)	56 (33.7%)
	R	7 (4.2%)	163 (98.2%)	4 (2.4%)	48 (28.9%)	84 (50.6%)	60 (36.1%)	43 (25.9%)	22 (13.3%)	147 (88.5%)	83 (50.0%)	110 (66.3%)
*Staphylococcus saprophyticus* (n = 35)	S	34 (97.1%)	0 (0.0%)	29 (82.9%)	29 (82.9%)	12 (34.3%)	14 (40.0%)	18 (51.4%)	25 (71.4%)	5 (14.3%)	10 (28.6%)	7 (20.0%)
	R	1 (2.9%)	35 (100%)	6 (17.1%)	6 (17.1%)	23 (65.7%)	21 (60.0%)	17 (48.6%)	10 (28.6%)	30 (85.7%)	25 (71.4%)	28 (80.0%)
*Staphylococcus aureus* (n = 15)	S	14 (93.3%)	0 (0.0%)	15 (100%)	12 (80.0%)	7 (46.7%)	9 (60.0%)	11 (73.3%)	13 (86.7%)	1 (6.7%)	7 (46.7%)	3 (20.0%)
	R	1 (6.7%)	15 (100%)	0 (0.0%)	3 (20.0%)	8 (53.3%)	6 (40.0%)	4 (26.7%)	2 (13.3%)	14 (93.3%)	8 (53.3%)	12 (80.0%)
*Micrococcus* spp. (n = 6)	S	5 (83.3%)	0 (0.0%)	6 (100%)	4 (66.7%)	3 (50.0%)	5 (83.3%)	4 (66.7%)	6 (100%)	0 (0.0%)	2 (33.3%)	1 (16.7%)
	R	1 (16.7%)	6 (100%)	0 (0.0%)	2 (33.3%)	3 (50.0%)	1 (16.7%)	2 (33.3%)	0 (0.0%)	6 (100%)	4 (66.7%)	5 (83.3%)
GPR spp. (n = 3)	S	3 (100%)	0 (0.0%)	3 (100%)	2 (66.7%)	0 (0.0%)	2 (66.7%)	3 (100%)	3 (100%)	0 (0.0%)	2 (66.7%)	0 (0.0%)
	R	0 (0.0%)	3 (100%)	0 (0.0%)	1 (33.3%)	3 (100%)	1 (33.3%)	0 (0.0%)	0 (0.0%)	3 (100%)	1 (33.3%)	3 (100%)
GNC spp. (n = 3)	S	3 (100%)	0 (0.0%)	2 (66.7%)	3 (100%)	0 (0.0%)	1 (33.3%)	1 (33.3%)	1 (33.3%)	0 (0.0%)	1 (33.3%)	0 (0.0%)
	R	0 (0.0%)	3 (100%)	1 (33.3%)	0 (0.0%)	3 (100%)	2 (66.7%)	2 (66.7%)	2 (66.7%)	3 (100%)	2 (66.7%)	3 (100%)
*Klebsiella aerogenes* (n = 2)	S	1 (50.0%)	0 (0.0%)	2 (100%)	0 (0.0%)	0 (0.0%)	0 (0.0%)	1 (50.0%)	1 (50.0%)	0 (0.0%)	1 (50.0%)	0 (0.0%)
	R	1 (50.0%)	2 (100%)	0 (0.0%)	2 (100%)	2 (100%)	2 (100%)	1 (50.0%)	1 (50.0%)	2 (100%)	1 (50.0%)	2 (100%)
*Klebsiella pneumoniae* (n = 2)	S	0 (0.0%)	0 (0.0%)	0 (0.0%)	2 (100%)	1 (50.0%)	0 (0.0%)	1 (50.0%)	2 (100%)	2 (100%)	2 (100%)	0 (0.0%)
	R	2 (100%)	2 (100%)	2 (100%)	0 (0.0%)	1 (50.0%)	2 (100%)	1 (50.0%)	0 (0.0%)	0 (0.0%)	0 (0.0%)	2 (100%)
*Enterobacter aerogenes* (n = 3)	S	2 (66.7%)	0 (0.0%)	2 (66.7%)	3 (100%)	1 (33.3%)	0 (0.0%)	1 (33.3%)	1 (33.3%)	3 (100%)	3 (100%)	0 (0.0%)
	R	1 (33.3%)	3 (100%)	1 (33.3%)	0 (0.0%)	2 (66.7%)	3 (100%)	2 (66.7%)	2 (66.7%)	0 (0.0%)	0 (0.0%)	3 (100%)
Total	S	221 (94.0%)	3 (1.3%)	221 (94.0%)	173 (73.6%)	106 (45.1%)	137 (58.3%)	163 (69.4%)	196 (83.4%)	30 (12.8%)	111 (47.2%)	67 (28.5%)
	R	14 (6.0%)	232 (98.7%)	14 (6.0%)	62 (26.4%)	129 (54.9%)	98 (41.7%)	72 (30.6%)	39 (16.6%)	205 (88.2%)	124 (52.8%)	168 (71.5%)

AMP = ampicillin, CHL = chloramphenicol, CIP = ciprofloxacin, CLN = clindamycin, CXM = cefotaxime, CXT = cefoxitin, DXT = doxycycline, E = erythromycin, GM = gentamicin, GNC = Gram-negative cocci, GPR = Gram-positive rods, K = kanamycin, R = resistant, S = susceptible, spp. = species, SXT = trimethoprim–sulfamethoxazole.

### 3.6. Multidrug resistance pattern of bacterial isolates

Of the 235 bacterial isolates tested, 181 (77.02%) were classified as MDR, while 25 (10.64%) were classified as XDR. The highest proportion of MDR was observed among *S saprophyticus* (94.29%), followed by *S aureus* (73.33%) and *S epidermidis* (70.6%). All isolates of *K aerogenes* (2/2, 100%), *K pneumoniae* (2/2, 100%), *E aerogenes* (3/3, 100%), Gram-positive rod spp. (3/3, 100%), and Gram-negative cocci spp. (3/3, 100%) were classified as MDR.

XDR was detected among *S epidermidis* (15/166, 9.03%), *S saprophyticus* (6/35, 17.14%), Gram-negative cocci spp. (2/3, 66.67%), *K aerogenes* (1/2, 50.00%), and *K pneumoniae* (1/2, 50.00%). No XDR isolates were detected among *S aureus*, *Micrococcus* spp., Gram-positive rod spp., or *E aerogenes*. No pandrug-resistant isolates were identified (Table 5).

**Table 5:** Multidrug-resistant, extensively drug-resistant, and pandrug-resistant profiles of bacterial isolates recovered from mobile phones among students and employees at Haramaya University College of Health and Medical Sciences, Harar, Eastern Ethiopia, 2022.

Bacterial isolate	Total tested	MDR n (%)	XDR n (%)	PDR n (%)
*Staphylococcus epidermidis*	166	121 (72.89)	15 (9.03)	0 (0.00)
*Staphylococcus saprophyticus*	35	33 (94.29)	6 (17.14)	0 (0.00)
*Staphylococcus aureus*	15	11 (73.33)	0 (0.00)	0 (0.00)
*Micrococcus* sp.	6	3 (50.00)	0 (0.00)	0 (0.00)
GPR sp.	3	3 (100.00)	0 (0.00)	0 (0.00)
GNC sp.	3	3 (100.00)	2 (66.67)	0 (0.00)
*Klebsiella aerogenes*	2	2 (100.00)	1 (50.00)	0 (0.00)
*Klebsiella pneumoniae*	2	2 (100.00)	1 (50.00)	0 (0.00)
*Enterobacter aerogenes*	3	3 (100.00)	0 (0.00)	0 (0.00)
Total	235	181 (77.02)	25 (10.64)	0 (0.00)

GNC = Gram-negative cocci, GPR = Gram-positive rods, MDR = multidrug-resistant, PDR = pandrug-resistant, sp. = species, XDR = extensively drug-resistant.

## 4. Discussion

Mobile phones are widely used worldwide and provide several benefits, particularly among university students and healthcare professionals who rely on them for communication and information access. However, frequent handling and close contact with users may allow mobile phones to harbor microorganisms and serve as potential fomites for microbial transfer.^[22,23]^

In the present study, Gram-positive bacteria accounted for 86.4% of the bacterial isolates and predominated over Gram-negative bacteria. This finding was comparable to a study conducted in Uganda (88.6%).^[17]^ The prevalence observed in the present study was higher than those reported from Jimma, Southwestern Ethiopia (71.2%),^[24]^ Nigeria,^[25]^ Yemen (70%),^[26]^ and Zambia 79%. Conversely, it was lower than those reported from Eastern Ethiopia^[16]^ (94.2%), Gondar University Hospital, Northwest Ethiopia (100%),^[18]^ Iran (99.2%),^[27]^ Alexandria, Egypt (100%),^[28]^ and India (100%).^[23]^ These variations may be explained by differences in study populations, environmental exposure, mobile phone handling practices, hand hygiene behaviors, and cleaning habits. In this study, we were able to isolate more Gram-positive bacteria than Gram-negative bacteria. This finding is higher than the study done in Jimma, Southwest Ethiopia,^[24]^ Gondar University Hospital, Northwest Ethiopia,^[18]^ and Uganda.^[17]^ The finding, however, is lower than the 2 reported from Iran,^[27]^ and Harar, Eastern Ethiopia.^[16]^ Gram-positive bacteria may survive longer under certain environmental conditions compared with some Gram-negative bacteria, and they are also common members of normal human skin flora, which may explain their frequent isolation from mobile phones.^[29]^ Because mobile phones are frequently handled and come into direct contact with users’ hands and faces, they may readily acquire microorganisms from the skin and surrounding environment.

The majority of the isolates in the current study were coagulase-negative staphylococci (CoNS), accounting for 85.5% of the isolates. This study’s findings were higher than the studies done in Jimma (54.5), south-west Ethiopia.^[24]^ Zambia (50%),^[30]^ Alexandria, Egypt (50%).^[28]^ However, the results of this research were lower than those of Iran (82.4%).^[27]^ The predominance of CoNS is consistent with their role as common colonizers of human skin and mucous membranes.^[31]^ Their frequent recovery from mobile phones may therefore reflect repeated transfer from users’ hands and skin to frequently handled phone surfaces. Although CoNS are commonly considered commensal organisms, some species can cause opportunistic infections, particularly among susceptible individuals and in healthcare settings.

The proportion of *S aureus* among the recovered bacterial isolates in the present study was 6.4% which was comparable with a study conducted in Sri Lanka (5%).^[32]^ However, higher proportions have been reported in studies conducted in Jimma,^[24]^ Gondar,^[15]^ Iran,^[27]^ Egypt, Malaysia (20.9%),^[33]^ and Brazil (32%).^[12]^ In contrast, a study conducted in Italy reported a slightly lower prevalence of 4%.^[34]^ The discrepancy could be explained by the fact that this bacterium is part of the normal flora of the skin and nose. Its prevalence as a bacterial contamination on mobile phones may be attributed to the ease with which it is discharged by a range of human activities such as sneezing, coughing, and other skin-contact-related actions.^[35]^

*K aerogenes* (1.3%) was the most commonly isolated Gram-negative bacterium. In our study, *Micrococcus* spp. were detected at a higher prevalence than most other bacterial isolates, although the proportion was lower than that reported in an earlier study (14.9%).^[17]^ *Micrococcus* spp. are widespread members of the normal skin and hand flora; however, they have also been associated with bloodstream infections, particularly in immunocompromised individuals.^[36]^

In the current study, Gram-negative rods, Gram-negative cocci, *P aeruginosa*, and *K pneumoniae* were identified among the Gram-negative bacterial isolates. These findings are consistent with those of previous studies, although the isolation frequencies differed. The presence of Gram-negative bacteria on mobile phones may indicate contamination with coliform-associated organisms and suggests the potential for fecal contamination of mobile phone surfaces.^[37]^

In this study, mobile phones belonging to male participants had nearly 4 times higher odds of bacterial contamination than those belonging to female participants (AOR: 3.92, 95% CI: 1.36–11.28). This finding is consistent with studies conducted in Iran,^[38]^ Mauritius,^[39]^ and Nigeria.^[25]^ Differences in hand hygiene, frequency of mobile phone use, occupational activities, and cleaning practices may contribute to variation in contamination between groups. Although differences in hygiene practices between men and women have been proposed as a possible explanation, such differences are not universal and should not be assumed to be solely responsible for the observed association. Other studies have reported no significant association between sex and mobile phone contamination or cleaning practices,^[40,41]^ suggesting that contextual and behavioral factors may influence this relationship. In addition, participants who did not regularly disinfect or clean their mobile phones had >7 times higher odds of bacterial contamination than those who cleaned their phones daily (AOR: 7.25, 95% CI: 2.16–24.34). This finding is biologically plausible because regular cleaning and disinfection may reduce the accumulation of microorganisms on frequently handled surfaces. The finding also emphasizes the potential importance of incorporating appropriate mobile phone hygiene into infection prevention and control education, particularly in health sciences and healthcare-related settings. Nevertheless, the cross-sectional nature of the study prevents establishing a causal relationship between cleaning practices and bacterial contamination.

Regarding awareness of bacterial contamination of mobile phones, participants who were unaware that mobile phones could be contaminated with bacteria had 4 times higher odds of bacterial contamination than those who were aware (AOR: 4.25, 95% CI: 1.29–13.97). This finding was consistent with a study conducted in Malaysia. Greater awareness may encourage users to adopt preventive behaviors, including regular cleaning and disinfection of mobile phones. However, awareness and cleaning practices may be interrelated, and the cross-sectional design does not allow the direction of this relationship to be established.^[41]^

Nowadays, resistance to 1 or multiple antimicrobials is the most serious health threat when treating patients.^[42]^ In the present study, gentamicin and chloramphenicol demonstrated the highest proportions of susceptible isolates (94.0% each), followed by clindamycin (83.4%), trimethoprim–sulfamethoxazole (73.6%), and erythromycin (69.4%). In contrast, the highest resistance rates were observed for ampicillin (98.7%), kanamycin (88.2%), cefotaxime (71.5%), and cefoxitin (54.9%). The high level of resistance to ampicillin observed in the present study is consistent with previous findings reported from Ethiopia.^[16]^ The detection of antimicrobial-resistant bacteria on mobile phones is of particular concern because frequently handled mobile devices may provide opportunities for the indirect transfer of resistant microorganisms. However, the detection of resistant organisms on mobile phones does not, by itself, establish bacterial transmission or infection.

## 5. Limitations

This study has some limitations. The cross-sectional design limits the ability to establish temporal or causal relationships. Self-reported information on mobile phone cleaning and hand hygiene may have been affected by recall or social desirability bias. The single-institution setting may limit the generalizability of the findings. Phenotypic bacterial identification may have limited species-level discrimination compared with molecular methods. Finally, the small number of some bacterial species limited the precision of species-specific antimicrobial susceptibility and MDR/XDR estimates.

## 6. Conclusion and Recommendation

This study demonstrated a high level of bacterial contamination of mobile phones among students and employees of Haramaya University College of Health and Medical Sciences. The predominant isolates were CoNS, *S aureus*, and Gram-negative bacteria, including *E coli* and *Klebsiella* spp. These findings suggest that mobile phones may serve as potential reservoirs for bacterial microorganisms and could facilitate their indirect transmission in healthcare-related environments. Regular cleaning and disinfection of mobile phones, together with improved awareness of hand hygiene and mobile phone hygiene among users, are recommended to reduce the potential risk of bacterial transmission.

## Acknowledgments

We are grateful to the Haramaya University College of Health and Medical Sciences Institutional Health Research Ethical Review Committee for providing ethical approval. We are grateful to the entire HU-CHMS administration and employee for their cooperation. Finally, we would like to express our heartfelt gratitude to the study participants for their contributions to this work.

## Author contributions

**Conceptualization:** Firayad Ayele, Tewodros Tesfa, Fitsum Weldegebreal.

**Data curation:** Kedir Urgesa, Shambel Mekonnen, Kasahun Bogale, Mohammed Ahmed, Sileshi Debebe, Fikru Tebeje, Haftu Asmerom, Fitsum Weldegebreal.

**Formal analysis:** Firayad Ayele, Shambel Mekonnen, Mesay Arkew, Tadesse Shume.

**Investigation:** Firayad Ayele, Shambel Mekonnen, Fikru Tebeje.

**Methodology:** Firayad Ayele, Kedir Urgesa, Shambel Mekonnen, Kasahun Bogale, Mohammed Ahmed, Sileshi Debebe, Fikru Tebeje, Haftu Asmerom, Mesay Arkew, Tadesse Shume, Tewodros Tesfa, Fitsum Weldegebreal.

**Project administration:** Fitsum Weldegebreal.

**Resources:** Firayad Ayele.

**Software:** Firayad Ayele, Shambel Mekonnen, Mesay Arkew.

**Supervision:** Firayad Ayele, Kedir Urgesa, Kasahun Bogale, Mohammed Ahmed, Sileshi Debebe, Fikru Tebeje, Haftu Asmerom, Mesay Arkew, Tadesse Shume, Tewodros Tesfa, Fitsum Weldegebreal.

**Validation:** Firayad Ayele, Haftu Asmerom.

**Writing – original draft:** Firayad Ayele.

**Writing – review & editing:** Firayad Ayele, Kedir Urgesa, Shambel Mekonnen, Kasahun Bogale, Mohammed Ahmed, Sileshi Debebe, Fikru Tebeje, Haftu Asmerom, Mesay Arkew, Tadesse Shume, Tewodros Tesfa, Fitsum Weldegebreal.
